# Effect of perception of sustainability in local food experiences on healthy eating tendency: mediator and moderator effects

**DOI:** 10.3389/fnut.2023.1150277

**Published:** 2023-06-07

**Authors:** Zühal Aksakallı Bayraktar, Serhan Oral, Samuray Hakan Bulut, Yusuf Bayraktar

**Affiliations:** ^1^Faculty of Tourism, Department of Gastronomy and Culinary Arts, Atatürk University, Erzurum, Türkiye; ^2^Faculty of Tourism, Department of Tourism Management, Atatürk University, Erzurum, Türkiye

**Keywords:** local food, social and environmental sustainability, healthy eating, food-related personality traits, online information search, process macro models

## Abstract

Tourists who favor local food typically care about healthy food choices. Their view of locally produced food as healthy is related to perceptions of sustainability. This relationship can be explained by tourists' personality traits and tendency to eat local food. This study aimed to establish the effect of tourists' perceptions of sustainability in the context of local food experiences on healthy eating tendencies. In addition, we aimed to determine the role of tourists' personality traits and local eating tendencies and elucidate the moderating role of searching online for information on food choices. An online questionnaire (via e-mail and WhatsApp) was used to obtain data from 379 research participants, recruited using a non-probabilistic sampling technique. A research model and hypotheses were formed based on Hayes PROCESS Macro models 90 and 6, and moderator and mediator effects were analyzed using these models. Healthy eating was well-explained by the model, and the perception of social and environmental sustainability in local food experiences (LFE-SES) positively affected food-related personality traits (FRPT), local food eating tendencies (LFET), and healthy eating (HE). While food-related personality traits did not mediate the relationship between the perception of sustainability and healthy eating, local food eating tended to mediate this relationship. In addition, when food-related personality traits and local food eating tendencies were evaluated together, they had a mediating role between the perception of sustainability and healthy eating. Searching online for information had an insignificant moderating effect. These findings help promote an understanding of healthy eating tendencies. Within the context of local food, they suggest critical theoretical and practical implications for the relationship between the perception of sustainability, food-related personality traits, local food eating tendencies, and healthy eating.

## Introduction

For years, policymakers have formulated regulations prioritizing consumer health over sustainability concerning food choices (1). Recently, the concept of sustainability – which encompasses social justice, environmental friendliness, and economic development – has been linked to the need for healthy food, and the topic of sustainable and healthy eating is now being studied more holistically (2–5). Though food consumption preferences currently prioritize health over sustainability, it is evident that consumer perceptions of sustainability have started to shift (6). This shift is so marked that environmental and social sustainability have moved from being phenomena influenced by consumers' tendency for healthy eating to becoming factors that drive such tendencies (7, 8).

Future food policies will continue to be influenced by research. Therefore, studies are needed to establish the link between consumers' perceptions of social and environmental sustainability and their tendency toward healthy eating. Alternative diets (9–13), organic foods (14, 15), and local food (16) are aspects highlighted individually or in combination in the growing body of relevant research (17, 18). However, a consensus has not been reached regarding the policies that establish the relationship between sustainability and healthy eating in the most accurate and objective way (1). This is because the social standing (19) and personality qualities (20) of the consumers who choose the food play a role in the relationship between sustainable and healthy food, in addition to the type of food chosen. Consumers who understand the connection between social and environmental sustainability and healthy eating are more likely to undertake their own research and seek information on controversial topics.

Food consumption is an increasingly significant sustainability issue because of its effects on human health, natural resources, and the social cohesion of communities (21). Modern food production and consumption are regarded as unsustainable (22), which have led to heightened consumer concerns about the environment and health. Knowledge of the origin of local foods and the transparency of local food chains have increased interest in local food (23). In addition, local foods are preferred for social sustainability reasons such as belonging, community, tradition, and loyalty (24).

The concept of sustainability is typically defined through social, environmental, and economic dimensions (25). However, our study focuses on the preference for healthy and local food linked to social and environmental sustainability. Increasingly, social and environmental sustainability are the basic dimensions used in scales examining human tendencies. In the present study, we tested a research model that assumes consumers who establish a relationship between sustainability and healthy eating develop this relationship through food-related personality traits and a tendency to eat local food. Further, the model contends that consumers' information-seeking behaviors play a role in this relationship. The literature shows that healthy nutrition is associated with many concepts, such as local food, alternative food, and food-related personality traits. Unlike many studies, our research model adopts a comprehensive perspective by considering the relationships between the variables. Testa et al. (25) focused on the relationship between the perception of social and environmental sustainability and healthy eating. They tested this relationship in the context of local food experiences. Thus, our research model mainly focuses on this relationship and aims to determine the role of variables in this context. Our study contributes to the literature by offering a holistic approach considering many variables affecting healthy eating. However, our study suggests that some of these variables can produce results contrary to expectations when considered in a model where other variables are also related, that is, where mediator and moderator effects occur.

Our study focused on tourists experiencing local food in Türkiye. It examined the impact of the findings on public and private institutions, policymakers, tourists, and businesses committed to the sustainability of local food. It has implications for advancing the understanding of the healthy eating tendencies of tourists in the context of local food.

### Theoretical background and literature review

Food consumption preferences and behavior are influenced by the healthiness of meals (1). The Theory of Reasoned Action explains individuals' behavior in the context of consumption, arguing that individuals' attitudes and subjective norms about an action affect their intentions and behaviors (26). While this theory explains situations under an individual's control, it falls short in situations they cannot control. Thus, the Theory of Planned Behavior emerged by adding perceived behavioral control as a variable in the model (27). According to the theory, individuals' attitudes, subjective norms, and perceived behavioral controls affect their intentions toward their behaviors.

The primary variable examined in our study was tourists' healthy eating behavior. In this context, the Theory of Planned Behavior forms the basis for explaining healthy eating behavior because individuals' approaches to sustainability affect their healthy eating behaviors (8, 28–30). In our study, this is linked to the “attitude” dimension of the Theory of Planned Behavior. Chen (31) emphasizes that consumers' personality traits related to food affect their intentions to buy organic food within the context of the Theory of Planned Behavior. Additionally, food-related personality traits reflect individuals' behavioral characteristics toward food (32–35). Therefore, it is assumed that personality traits explain healthy eating behavior in individuals, and tendencies to eat local food can also affect healthy eating behavior (36–40). Within this context, a hypothetical model was created, taking into account the Theory of Planned Behavior and assuming that tourists' healthy eating behavior in their local food experiences can be explained by sustainability attitudes, personality traits, and tendency to eat local food.

### Effect of perception of social and environmental sustainability in local food experiences on healthy eating

COVID-19 caused people to reflect on their actions and motivations and become more conscious of issues related to the environment, sustainability, ecosystem balance, and human health (41). At the same time, studies of local dishes frequently concentrate on cultural elements, environmental factors, and healthy eating (42). Consuming sustainable, healthy, and local products favorably impacts environmental preservation and the sustainability of food production systems. It may also result in many statistically significant changes in behavioral attitudes that support sustainability (41). For instance, environmental concerns may influence gastronomy tourists' travel intentions (43), and tourists might decide whether to eat local foods based on their perceived safety and health (44–46). A global transformation in eating paradigms is anticipated due to the attempts to provide healthy and environmentally sustainable diets by 2050 (47). The EAT-Lancet Commission's model for sustainable eating (29) asserts that diets high in plant foods and low in animal products reduce adverse environmental effects (48, 49). Further, the 49 define healthy eating in the context of sustainability as an approachable, affordable, safe, equitable, and culturally acceptable diet that supports people's health in all of its dimensions and has minimal impact on the environment. In terms of its effects on the ecosystem, sustainable healthy eating fosters the preservation of biodiversity. At the same time, it adopts and respects the values related to local culture and culinary practices, knowledge about food and consumption patterns, and the fair acquisition, production, and consumption of food in terms of its socio-cultural effects (30).

Healthy eating and living are socially valued, and this awareness can be converted into long-term behavior (50). Sweden's official diet guidelines characterize the “ideal eater” as someone who enjoys fresh, healthy, and varied foods while prioritizing sustainability – thus, demonstrating that policymakers also respect this assessment (51). Having a sustainable lifestyle impacts healthy eating choices in a positive way (7). Donato et al. (8) discovered that customers perceive goods in sustainable packaging as being healthier, suggesting a link between healthy eating and perceptions of social and environmental sustainability. This led to the establishment of hypothesis H_1_:

*H*_1_*: The perception of social and environmental sustainability in local food experiences positively impacts healthy eating*.

### Effect of perception of social and environmental sustainability in local food experiences on food-related personality traits

Tourists' experiences with local food bring economic, cultural, and environmental sustainability to destinations (52–54). The sustainability dimension that the local food experience brings to destinations also affects tourists' consumption motivations. Kline et al. (55) stated that environmental sustainability concerns affect consumers' motivation to experience local food, and environmental and social sustainability motivations affect consumption choices Hashem et al. (56). Testa et al. (25) suggested that several motivations affect tourists in rural tourism destinations and that social and environmental sustainability should be considered as key motivators for explaining local food and beverage consumption.

Food-related behaviors and consumption preferences are affected by many individual characteristics, including important psychological variables (35). These features are explained with the concept of “food-related personality traits” (57–59). Studies have revealed that food-related personality traits play an important role in influencing tourists' food consumption behavior (32, 60, 61).

To fully comprehend the personality traits associated with food and observed in visitors, it is crucial to ascertain why certain foods are liked (62). Similarly, people's perspectives on social and environmental sustainability should be assessed. Hopwood et al. (63) examined the connection between individual motivations and sustainability behavior in the travel industry. They discovered a substantial correlation between motivational incentives reflecting personality factors and sustainability behavior. Consumption patterns of organic foods, associated with perceptions of social or environmental sustainability, are clearly correlated with personality characteristics (64). This knowledge suggests a relationship between food-related personality factors and perceptions of social and environmental sustainability in local food experiences. This presumption led to the creation of hypothesis H_2_.

*H*_2_*: The perception of social and environmental sustainability in local food experiences positively impacts food-related personality traits*.

### Effect of perception of social and environmental sustainability in local food experiences on local food eating tendencies

People want to try local dishes for many personal reasons, including their view of sustainability (65), and the consumption of products produced locally and sustainable behavior are related (66). The literature analyzes ideas about sustainability and locally grown food from multiple viewpoints. Hashem et al. (56) emphasized the issue of environmental sustainability, and that local food is more environmentally sustainable. Alsetoohy et al. (67) evaluated local food from a social perspective, suggesting that purchasing local food products is an important sustainable practice.

Local food is more likely consumed by those concerned about social, economic, and ecological justice (68). One consumer motivation leading to the rise in demand for local food is the favorable association between regional foods and socially and environmentally responsible food production (69). Along with the common sustainable practice of purchasing local food (70), the sustainability of the natural environment is a common concern (71). Analyses of the attraction of food tourism indicate that local food is associated with three basic concepts, one of which is sustainability (72). Based on these studies, the H_3_ hypothesis was developed:

*H*_3_*: The perception of social and environmental sustainability in local food experiences positively impacts local food eating tendencies*.

### Effect of food-related personality traits on local food eating tendencies

For international tourists, local cuisine provides a new experience and creates opportunities to be involved in local cultures (73). Thus, local cuisine is an attractive factor for a tourism destination (72). However, only some tourists eat local food. Baah et al. (74) asserted that personality traits play an important role in this context – while some travelers are curious to try out new cuisines, others are apprehensive about unusual or unfamiliar ingredients. Instead of normative attitudes (beliefs about what one thinks other people should do), personal behavioral characteristics determine whether tourists intend to try local food (75). Food-related personality traits are personal elements that can influence the desire to eat local food (76). These traits describe how people behave in relation to food and include psychological factors that influence tourists' food consumption (32–35). Concepts like neophilia, neophobia, and food involvement emerge in the literature when local food eating habits and personality factors are integrated.

Food neophilia makes people more likely to consume local food and travel in search of new food experiences (28, 33, 61). Visitors with food neophiliac behavior show a greater tendency to seek out and experience new foods (35). Similarly, neophobic personality traits affect tourists' local food consumption tendencies (33, 77). A neophobic tendency negatively affects acceptance of local cuisine in terms of cognitive, sensory, and conative aspects (74, 78, 79). Choi and Jeon (80) compared the factors affecting Chinese and Japanese tourists' tendencies to consume local food and found that neophobic personality traits influence Chinese tourists' consumption of local food. Hussain et al. (81) found that food neophobia had a negative (and food neophilia a positive) moderator role between tourists' attitudes toward local foods and their intention to try them. Tasting new flavors and meeting people of new cultures in ethnic restaurants is an important motivation for neophiles, and not for those who are neophobic (82). Tourists with high food involvement – another food-related personality trait – tend to eat local food (28). In addition, tourists' preference for local food increased during festivals where food is presented in ways that highlight its nutritional value (83). The H_4_ hypothesis was developed based on the evaluation of this knowledge, assuming a strong relationship between tourists' tendency to consume local food and beverages and their food-related personality traits (84):

*H*_4_*: Food-related personality traits positively impact local food eating tendencies*.

### Effect of food-related personality traits on healthy eating

To fully comprehend the food-related personality traits visitors demonstrate, it is critical to determine why certain foods are favored (62). Steptoe et al. (85) found nine main reasons people choose certain foods, one of which is health. Eating habits vary depending on the priority placed on maintaining a healthy lifestyle. Individuals with high health recognition are more interested in local foods considered good for human health and the environment (86) and functional and organic foods (87). In contrast, a person's relationship with food depends on various factors related to their eating habits. Food choices can be directly influenced by food involvement, which may also indirectly influence other factors known to affect choice, such as anticipation, hedonism, or eating place and time (88). Food involvement appears to be associated with overall nutritional health linked to these potential effects (89). People with a high level of food involvement in their food-related personality traits show healthy eating and drinking behaviors, such as fruit and vegetable consumption or a tendency to eat less fat, compared with others (31, 90–92). Additionally, individuals who do not exhibit neophobic personality traits (93) and who have high food involvement accept organic food more readily because of its advantages for both human health and the environment and, as a result, eat more healthily (94). Neophobic consumers tend to be less willing to try new foods (31). The literature suggests a strong correlation between a person's food-related personality traits and their propensity for healthy eating (95). The H_5_ hypothesis was developed as a result of this analysis.

*H*_5_*: Food-related personality traits positively impact healthy eating*.

### Effect of local food eating tendencies on healthy eating

Consumers buy local foods because they perceive these products as healthy, natural, supportive of animal welfare, having sensory appeal, and being well-priced (96). The “localness” of food represents environmental sustainability, better taste, and healthier food sensations for consumers (38). Those who favor local foods believe their consumption promotes health and boosts community sustainability by supporting local businesses (97). There is also compelling evidence that consumers associate local foods with freshness, quality, nutritional content, dependability, local flavors, naturalness, being healthy, and being good for the environment. In recent years, consumers have actively sought information about their food choices, selecting food based on high-quality ingredients and nutritional values. For example, European consumers increasingly prefer healthy and sustainable foods, and consumers are generally more health-conscious and prefer locally sourced, fresh, and additive-free foods (69). “Locavores” (98) actively seek out local food because they believe it to be more wholesome, flavorful, nutritious, and sustainable. They take pride in their local food choices and are interested in learning more about the origins of their food and the relationship between nutrition and health (40).

Individuals generally perceive local foods as fresh, healthy, and of better quality (39). Salois (37) found that local food outlets promote better dietary choices and healthier eating habits. Similarly, Little et al. (36) demonstrated that buying local products leads consumers to adopt healthier eating styles. Based on the knowledge that local foods are perceived as healthier, we consider the relationship between local food and healthy eating significant (99), leading to the H_6_ hypothesis.

*H*_6_*: Local food eating tendency positively impacts healthy eating*.

### Moderator effect hypotheses

The key component influencing the tendency toward healthy eating is knowledge (100). The Internet is a popular source of knowledge regarding healthy eating (101–103), and Mete et al. (104), for example, analyzed the rise in interest in healthy eating blogs. Trust in online sharing may also impact food consumption behaviors (105). People use social media to obtain information about healthy eating, and social networks influence how people look for and select products and services (106). Adults use social media as an information source for many topics, including dietary preferences and healthy eating (107–109). Social media is one of many elements influencing nutrition because it is viewed as a significant source of information regarding eating options (110, 111). Its use frequently raises the healthy eating recognition (112), and social media users' constructive interactions can encourage healthy eating (113). In research on nutritional information-seeking activities and how they relate to food consumption in China, Wang et al. (114) found that people who were more interested in food knowledge typically ate more healthily. When switching to a healthy diet, online information was often given great consideration (115). Thus, hypotheses H_7_ and H_8_ were developed.

*H*_7_*: Online information searching has a moderating role in the effect of perception of social and environmental sustainability in local food experiences on healthy eating*.
*H*_8_*: Online information searching has a moderating role in the effect of local eating tendencies on healthy eating*.

### Mediator effect hypotheses

Food consumption is related to several environmental effects, and consumer food choices are affected by environmental decisions (116). The issue of sustainability and healthy food has also become important for public policy and academic research because it can potentially reduce current environmental and health problems (117). Food consumption is an important driver of environmental pressure, and thus, adopting healthy eating approaches is environmentally friendly and a beneficial option for human health (118, 119). While organic foods are often regarded as more healthy, natural, nourishing, and sustainable than industrial foods (31), consumers are often less sure about whether the local food supplied to them is a better environmental choice than non-local options (120). Lazzarini et al. (121) argued that customers evaluate local products favorably in terms of social and environmental sustainability.

Numerous studies have investigated the connection between sustainable behaviors and tendencies toward healthy eating (7, 8, 41, 42), and research has also considered how these variables relate to personality traits. According to Bergman et al. (51), people who prioritized sustainability ate well and had high levels of cultural capital. Cultural capital (122) is a trait that plays a part in pursuing healthy eating and sustainable behavior. Food neophobia, another personality trait, should also be taken into consideration, according to Rabadan et al. (20), who emphasized the development of innovative techniques for healthy and sustainable food production. Food neophobia is a barrier to accepting edible insect consumption, a topic of several studies on sustainable alternative food and healthy eating (123, 124). Zarba et al. (125) suggested that eating seaweed (algae) as part of a sustainable food strategy promoted health – a widespread practice in traditional European cuisine. Custodio et al. (126) proposed that the approach used for seaweed in European culture could also prevent neophobic reactions in adopting halophyte crops, highlighting the connection between personality factors and a tendency toward healthy eating and sustainable food consumption.

The relationship between social and environmental sustainability and healthy eating has been investigated through alternative foods and diets (127). Alternative foods include *in-vitro* meats that reduce animal-based, plant-based, insect-based, and non-meat dietary proteins (10). The creation of alternative foods reflects the rising demand for sustainable and nutritious eating. However, not all affluent consumers are willing to eat this type of food (20). Consumers with high neophobic characteristics refused to taste insect-based foods, even after being informed about their ecological benefits for environmental sustainability through reducing land and water use, ammonia and greenhouse gas emissions, and increasing feed conversion rates (128). de Boer et al. (129) stated that red meat consumption could be reduced by switching to a plant-based diet and eating fish, but individuals with low food involvement may not accept fish as a protein source (130). Similarly, individuals who follow a vegetarian diet typically understand social and environmental sustainability, consider health risks, and have personality traits that make them open to new experiences (131). Thus, some personality-based qualities play a role in the relationship between sustainable behavior and healthy eating tendency. Therefore, the H_9_ hypothesis was developed.

*H*_9_*: Food-related personality traits have a mediating role in the effect of perception of social and environmental sustainability in local food experiences on healthy eating*.

Given the growing interest in food tourism, the places where local food is produced must have environmentally friendly and sustainable attributes (132). Local food is thought to help local economies and has a smaller social carbon footprint than food produced in a traditional manner, making it healthier and more environmentally friendly (133). Even with traumatic events that threaten global health – such as the COVID-19 pandemic – consumer trust in local food based on health and sustainability has not been shaken (134). Individuals who prefer restaurants offering locally produced food have higher sustainability and healthy eating tendencies than those who prefer fast food (135). People are looking for environmentally friendly, safe, and healthy foods as the desire to eat locally grows. In recent years, Google searches for “clean food” have increased by 52%, “local food” by 20%, “safe food” by 31%, and “healthy food” by 30% (136). These trends highlight the significance of simultaneous consideration of sustainable behavior, a tendency toward healthy eating, and a desire to consume local foods. Eating locally demonstrates a person's commitment to sustainable consumption practices and adoption of healthy eating (137). Zakowska-Biemans et al. (117) focused on sustainable and healthy eating, arguing that local food choice was a variable that affected these concepts. Therefore, local food can offer a practical way of eating healthily within the social and environmental sustainability framework. In this context, three hypotheses were further developed.

*H*_10_*: Local food eating tendency has a mediating role in the effect of perception of social and environmental sustainability in local food experiences on healthy eating*.
*H*_11_*: Local food eating tendency has a mediating effect, and online information searching has a moderating effect on the conditional indirect effect of the perception of social and environmental sustainability in local food experiences on healthy eating*.
*H*_12_*: Food-related personality traits and local food eating tendencies have a mediating role in the effect of perception of social and environmental sustainability in local food experiences on healthy eating*.

## Materials and methods

### Research model and hypotheses

**Figure 1:**
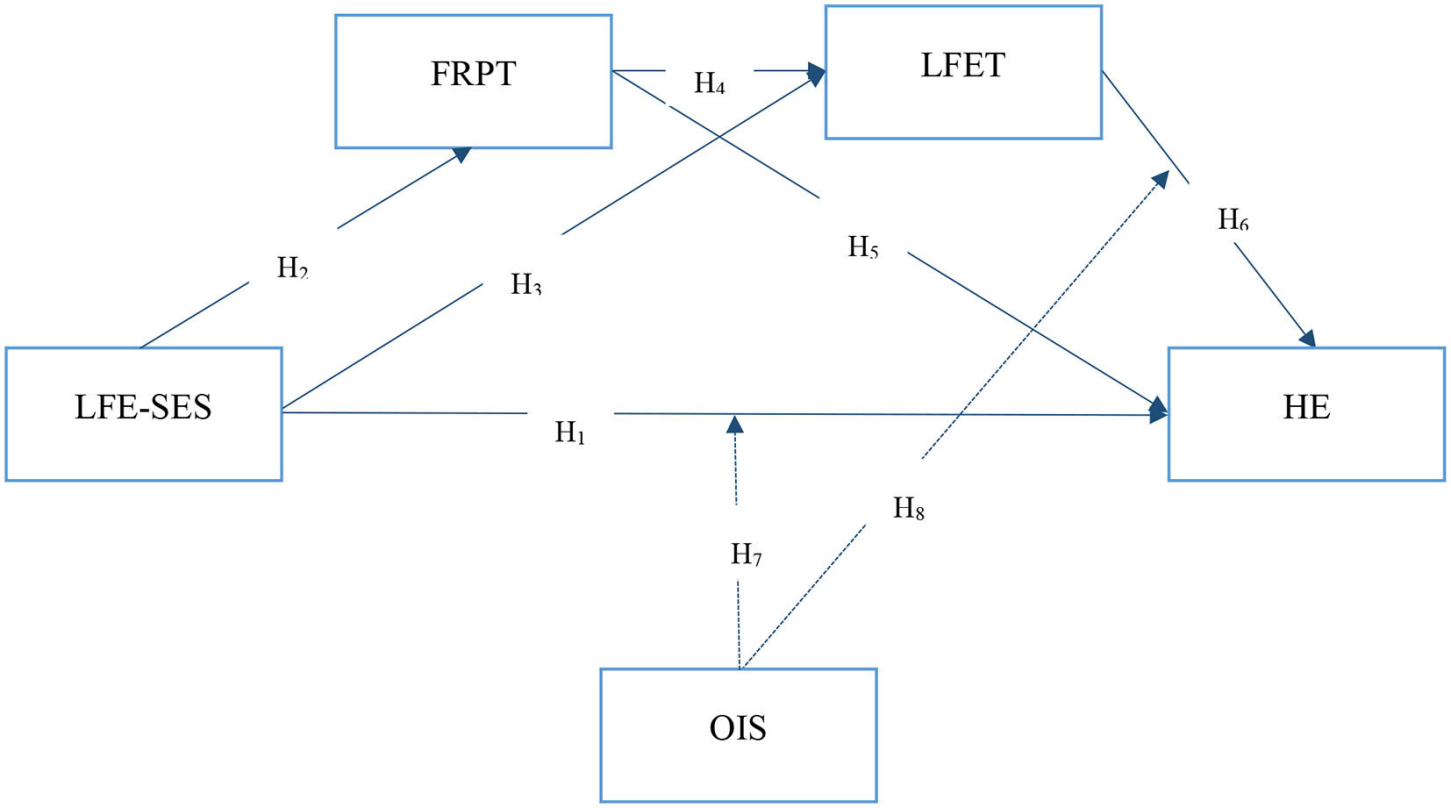
Research model (Hayes model 90). LFE-SES, Social and Environmental Sustainability in Local Food Experiences; FRPT, Food-related Personality Traits; LFET, Local Food Eating Tendency; HE, Healthy Eating; OIS, Online Information Research.

The research model was designed based on the Hayes (138) PROCESS Macro (model 90) shown in Figure 1. Hayes PROCESS Macro is a structural equation-based (SEM) analysis technique.

### Sample and data collection

The study was approved by Atatürk University Social and Human Sciences Ethics Committee (Ref: 2023:1–14). The questionnaire created for data collection was examined by three field experts who ensured that the language use and expression characteristics of the scales were appropriate. The questionnaire form was created using Google Forms and delivered to the participants via e-mail and WhatsApp. The form consisted of two parts. The first part comprised four demographic questions and a single question about online information search. In the second part, there were scale questions consisting of 26 items covering the four variables in the study. The research population was all tourists who have experienced local food in Türkiye, and an appropriate non-probabilistic sampling technique was used to represent it. Field Hair et al. (139) proposed that at least ten respondents should be used for each parameter in a scale to test the measurement model. Twenty-six parameters were included in all the scales used in our research, and 425 participants completed the questionnaire – a sample size that had the power to represent the population. From the collected data, 379 questionnaires were suitable for further analysis and deemed sufficient to test our research model. Participants were informed about the research at the outset of data collection, and all took part voluntarily.

### Measures

The variables included in the research model were measured using scales with proven validity and reliability in the literature. These included: the perception of social and environmental sustainability in local food experiences (LFE-SES), food-related personality traits (FRPT), local food eating tendency (LFET), and healthy eating (HE). All the variables comprised one dimension apart from food-related personality traits, which consisted of the two dimensions of food neophobia and food involvement. The food neophobia dimension was measured with six items, and the food involvement dimension with six items. Responses used a Likert scale from 1 (Strongly Disagree) to 5 (Strongly Agree). To measure the online information search status of the participants, the statement “*I do online searches about whether local food is healthy or not”* was added to the questionnaire, requiring a categorical response in the form of *Yes* or *No*. The sources of the scales and other details are given in Table 1.

**Table 1:** Scales used in the research.

**Scale name**	**Dimensions**	**Items**	**Author**
Social and environmental sustainability in local food experiences (LFE-SES)	1	7	(25)
Food-related personality traits (FRPT)	2	12	(31)
Local food eating tendency (LFET)	1	4	(140)
Healthy Eating (HE)	1	3	(25)

### Reliability and validity

This study aimed to determine the mediating effect of food-related personality traits and local food eating tendencies on the effect of social and environmental sustainability perception on healthy eating in local food experiences and the moderating effect of online information search. To achieve this aim, a measurement model test was conducted using AMOS v20 (IBM SPSS: Chicago) to reveal the relationship and harmony between the variables in the study. The measurement model results are shown in Table 2.

**Table 2:** Measurement model results.

	**x^2^**	**df**	**x^2^/df**	**SRMR**	**RMSEA**	**GFI**	**AGFI**	**CFI**	**TLI**	**Multivariate**
Measurement Model 1	939,159	291	3,227	0.812	0.077	0.822	0.785	0.855	0.838	18,755
Measurement Model 2	693,864	288	2,409	0.755	0.061	0.869	0.840	0.909	0.897	

The measurement model determined that the dataset had a normal distribution (Multivariate: 18,755) (141), but the fit indices were not at an acceptable level (model 1) (139, 142–144). Thus, modification indices were examined. A first modification was made between the item “*I would like to participate in local cuisine training”* and “*I would like to prepare the local dishes I tried once I return from the places I visited”* in the LFET scale. Second, in the LFE-SES scale, a modification was made between the items “*Eating local food helps me to be in solidarity with local producers”* and “*Eating local food allows me to contribute to the local economy”*. Finally, in the FRPT scale, a modification was made between the items “*Deciding what to eat is more important compared to other daily decisions”* and “*I think too much about what to eat every day”*. The fit indices were acceptable (model 2) after the modifications (139, 142–144). The visuals of the measurement models are presented in the Supplementary material. The Average Variance Extracted (AVE), Composite/Construct Reliability (CR), and Cronbach's Alpha values were examined to assess the validity and reliability of the scales used in the study. The results are presented in Table 3.

**Table 3:** AVE, CR, and Cronbach's alpha values.

**Scale**	**#Items**	**AVE**	**CR**	**Cronbach's Alpha**
LFE_SES	7	0.451	0.846	0.835
LFET	4	0.384	0.709	0.753
FRPT	12	0.465	0.909	0.861
HE	3	0.509	0.754	0.743

AVE, Average Variance Extracted; CR, Composite/Construct Reliability.

For the reliability of the structure, CR and Cronbach's Alpha values were examined, and values for each variable were above 0.70, indicating a high level of reliability (145, 146). We considered AVE values to test the construct validity of the scales. These were also within acceptable limits (145, 147).

### Discriminant validity

The Fornell-Larcker criterion test, Cross Loadings, and Heterotrait-Monotrait (HTMT) Ratio of Correlations are used to determine discriminant validity (147). Of these techniques, HTMT is considered the most inclusive and less restricted (148). Although HTMT is recommended for least squares path modeling, it can also be applied in SEM (148, 149). Thus, the HTMT criterion proposed by Henseler et al. (150) was used to determine discriminant validity in the study. Henseler et al. (150) define HTMT as the ratio of the mean of the correlations of the items of the variables to the geometric mean of the correlations of the expressions of the same variable (monotrait-heteromethod correlations). In this study, HTMT values were calculated with the AMOS v26 extension created by Gaskin (151). Henseler et al. (150) have indicated that the distance between the variables in the HTMT criterion should be below.85. The HTMT values of the research variables in this study are given in Table 4.

**Table 4:** Discriminant validity (HTMT criterion).

	**LFE_SES**	**LFET**	**HE**	**FRPT**
LFE_SES	1			
LFET	0.651	1		
HE	0.746	0.588	1	
FRPT	0.474	0.714	0.410	1

The HTMT criterion used to determine the discriminant validity in the study revealed that all values between the variables were acceptable (HTMT < 0.85). Thus, the discriminant validity of the study was demonstrated.

### Normal distribution

To examine the distribution of the variables in the dataset, the arithmetic mean, mode, and median values were analyzed, aiming for the mode and median values to be within ±1 range from the arithmetic mean. The mode (3.86), median (3.85), and arithmetic mean (3.86) for LFE-SES were within the reference range based on Pallant (152). Similarly, the values for the other variables were also acceptable (FRPT – mode: 4.00; median: 3.75, and arithmetic mean: 3.73; LFET – mode: 4.00; median: 4.00, and arithmetic mean: 3.83; HE – mode: 4.00; median: 3.66, and arithmetic mean: 3.65). Skewness and kurtosis were also examined to assess the normal distribution of the dataset, and ±1.96 was accepted as the reference range (152). The skewness (−0.822) and kurtosis (1.299) values for LFE-SES, FRPT (skewness:−0.630; kurtosis: 1.458), LFET (skewness: −0.406; kurtosis: 0.404), and HE (skewness: −0.459; kurtosis: 0.130) all indicate that the dataset followed a normal distribution. Additionally, histogram graphics were examined to confirm the normal distribution, and the multivariate value (18.755) in Table 2 also supports the normal distribution of the dataset.

## Results

### Descriptive statistics

Descriptive information about the 379 participants is shown in Table 5.

**Table 5:** Descriptive statistics (*N* = 379).

**Trait**	**Variable**	***n***	**%**
Gender	Woman	207	54.6
	Man	172	45.4
Age	18–26 years	82	21.6
	27–36 years	111	29.3
	37–46 years	108	28.5
	47 years and over	78	20.6
Education status	Secondary education	13	3.5
	Associate degree	24	6.3
	Bachelor's degree	137	36.1
	Graduate	205	54.1
Monthly income	Minimum wage 10.000 TL	48	12.7
	10.001 TL – 15.000 TL	82	21.6
	15.001 TL and over	175	46.2
Online information search	Yes	180	47.5
	No	199	52.5

More than half the participants (54.6%) were women. The 37-46-year-old age group had the highest participation (28.5%), while the 47 years and older group (20.6%) was the smallest group. The highest education level was postgraduation (54.1%), and the lowest was secondary education (3.5%). Almost half the sample (46.2%) had an income of 15,000 TL and above, while 12.7% received 10,000 TL or less. While 47.5% of the participants answered “Yes” to the question on online research regarding local food, 52.5% indicated that they did not do this research.

### Hypothesis testing

The research was designed to examine the role of food-related personality traits, the mediating role of local food eating tendencies, and the moderating role of online information searching in the effect of social and environmental sustainability perception on healthy eating in local food experiences. The research model and hypotheses were shaped through SEM, in line with the purpose of the research. Hayes PROCESS Macro is an SEM analysis technique using SPSS PROCESS v4.2 to test the study model. The study hypotheses were tested using the 90th model of Hayes (138). To ascertain the link between the study variables, a correlation analysis was carried out before the hypotheses testing. Table 6 displays the findings of the correlation analysis.

**Table 6:** Mean value, standard deviation, and correlation of all variables (*N* = 379).

**Variable**	**M**	**SD**	**1**	**2**	**3**	**4**
1. LFE-SES	3.866	0.611	1			
2. FRPT	3.730	0.592	0.387^**^	1		
3. LFET	3.831	0.689	0.494^**^	0.555^**^	1	
4. HE	3.655	0.732	0.595^**^	0.320^**^	0.424^**^	1

*N* = 379, ^**^p < 0.001.

The correlation analysis indicated a statistically significant relationship between all variables. A significant positive relationship was determined between LFE-SES and FRPT (*r* = 0.387, *p* < 0.001), LFET (*r* = 0.494, *p* < 0.001), and HE (*r* = 0.595, *p* < 0.001). Additionally, there was a substantial and positive correlation between FRPT and HE (*r* = 0.320, *p* < 0.001) and LFET (*r* = 0.555, *p* < 0.001). Finally, a substantial and positive connection was discovered between LFET and HE (*r* = 0.424, *p* < 0.001). Overall, significant and favorable associations between all study variables were found. To evaluate the study hypotheses, we used the Hayes (138) PROCESS Macro model 90, based on SEM, and carried out using SPSS PROCESS v4.2. Table 7 displays the results of the hypotheses testing.

**Table 7:** Hayes model 90 hypotheses test results.

**No**	**Hypothesis**			**β**	**LLCI**	**ULCI**	***p***	***R* ^2^**	**Result**
H_1_	LFE-SES → HE			0.70	0.3281	1.0815	0.000	0.38	Supported
H_2_	LFE-SES → FRPT			0.37	0.2846	0.4658	0.000	0.14	Supported
H_3_	LFE-SES → LFET			0.37	0.2749	0.4670	0.000	0.39	Supported
H_4_	FRPT → LFET			0.49	0.3977	0.5958	0.000	0.39	Supported
H_5_	FRPT → HE			0.03	−0.0896	0.1569	0.591	0.38	Not supported
H_6_	LFET → HE			0.18	−0.1461	0.5174	0.271	0.38	Not supported
H_7_	LFE-SES → OIS → HE^*^			−0.07	−0.2988	0.1580	0.544	0.38	Not supported
H_8_	LFET → OIS → HE^**^			−0.01	−0.2173	0.1829	0.865	0.38	Not supported
H_9_	LFE-SES → FRPT → HE			0.01	0.0240	−0.0309	-	-	Not supported
H_11_	LFE-SES → LFET → HE^***^	OIS	1	0.06	0.0096	0.1222	-	-	Not supported
			2	0.05	−0.0027	0.1210	-	-	

95% CI, *p* < 0.001, ^*^Int_1: OIS: Moderator, ^**^Int_2: OIS: Moderator, ^***^Moderated Mediator Effect (Conditional Indirect Effect).

The research model and hypotheses were tested with the Bootstrap technique based on SEM and the Hayes (138) 90th model with a 95% confidence interval. The effect of LFE-SES on HE (H_1_) was significant and positive (β = 0.70; *p* < 0.001; LLCI: 0.3281; ULCI: 1.0815); the effect on FRPT (H_2_) was significant and positive (β = 0.37; *p* < 0.001; LLCI: 0.2846; ULCI: 0.4658), and its effect on the LFET (H_3_) was also significant and positive (β = 0.37; *p* < 0.001; LLCI: 0.2749; ULCI: 0.4670). In addition, while the effect of FRPT on LFET (H_4_) was significant and positive (β = 0.49; *p* < 0.001; LLCI: 0.3977; ULCI: 0.5958), the effect on HE (H_5_) was not statistically significant (β = −0.03; *p* > 0.05; LLCI: −0.0896; ULCI: 0.1569). The effect of LFET on HE (H_6_) was also not significant (β = 0.18; *p* > 0.05; LLCI: −0.1461; ULCI: 0.5174).

Based on the research model, the moderating role of OIS in the effect of LFE-SES on HE was examined (H_7_), and it was not found to be significant (β = −0.07; *p* > 0.05; LLCI: −0.2988; ULCI: 0.1580). In addition, the moderating role of OIS in the effect of LFET on HE was insignificant (β = −0.01; *p* > 0.05; LLCI: −0.2173; ULCI: 0.1829). When the mediator role of FRPT in the effect of LFE-SES on HE was examined (H_9_), it was found insignificant (β = 0.01; *p* > 0.05; LLCI: 0.0240; ULCI: −0.0309). The moderated mediator effect (conditional indirect effect) of LFE-SES on HE was evaluated based on the study model, and OIS served as a moderator in the hypothesis that LFET is a mediator (H_11_). The moderated mediator effect of LFE-SES on HE –where LFET was the mediator and OIS the moderator – turned out to be insignificant (OIS:1 = 0.06; LLCI: 0.0096; ULCI: 0.1222; OIS:2 = 0.05; LLCI: −0.0027; ULCI: 0.1210) as a result of the previously mentioned conditional indirect effect.

**Figure 2:**
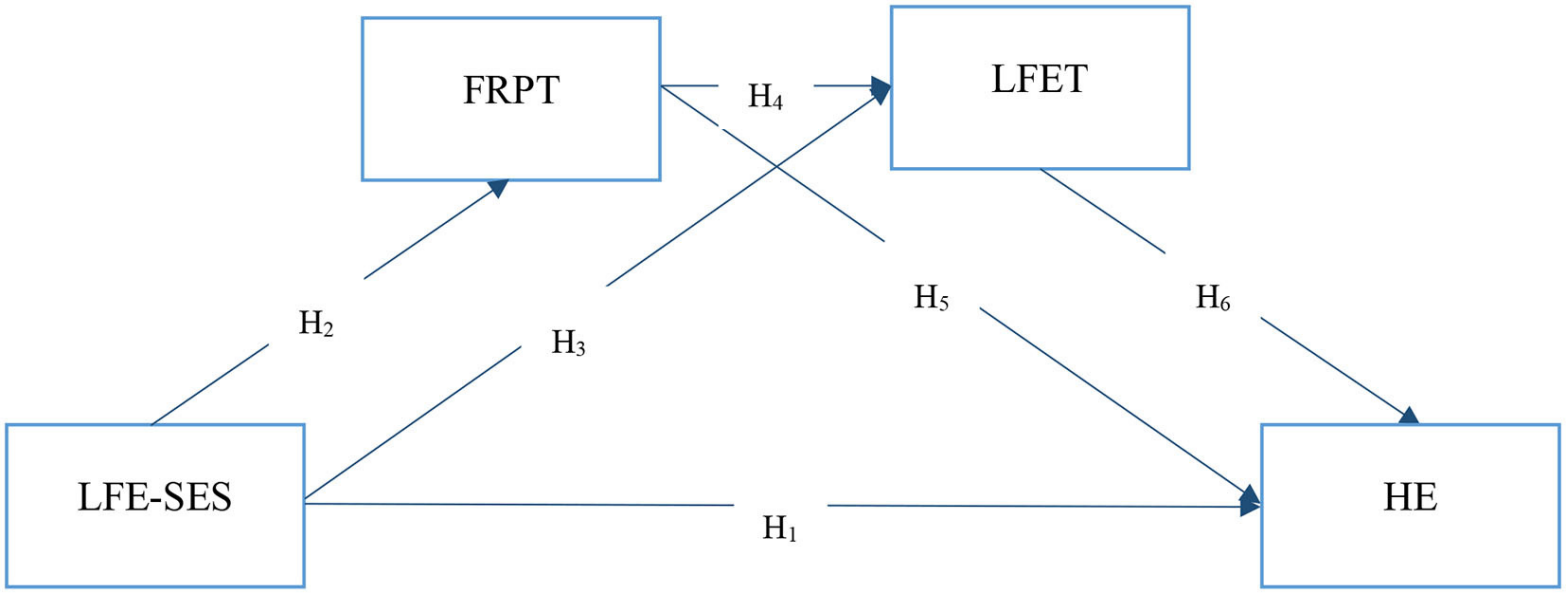
Research model [Hayes model 6 (2 mediators)].

The SPSS PROCESS v4.2 investigation did not produce any significant results regarding the moderating role of OIS using model 90. There were strong theoretical arguments for including OIS in our model. However, the moderating role of OIS was not significant, and it may have negatively affected the model fit. Therefore, we removed the OIS variable from the model and repeated the hypotheses testing. After removing OIS, the research model was consistent with Hayes (138) PROCESS Macro model 6 (2 mediators), and the model was reanalyzed using the framework shown in Figure 2.

The results of the hypotheses tests that were repeated based on the research model and tested in the context of Hayes (138) model 6 (2 mediators) are shown in Table 8.

**Table 8:** Hayes model 6 hypotheses test results.

**No**	**Hypothesis**	**β**	**LLCI**	**ULCI**	***p***	***R* ^2^**	**Result**
H_1_	LFE-SES → HE	0.60	0.4914	0.7150	0.000	0.37	Supported
H_2_	LFE-SES → FRPT	0.37	0.2846	0.4658	0.000	0.14	Supported
H_3_	LFE-SES → LFET	0.37	0.2749	0.4670	0.000	0.39	Supported
H_4_	FRPT → LFET	0.49	0.3977	0.5958	0.000	0.39	Supported
H_5_	FRPT → HE	0.04	−0.0707	0.1702	0.417	0.37	Not supported
H_6_	LFET → HE	0.16	0.0529	0.2727	0.003	0.37	Supported
H_9_	LFE-SES → FRPT → HE^*^	0.01	−0.0256	0.0675	-	-	Not supported
H_10_	LFE-SES → LFET → HE^**^	0.06	0.0178	0.1091	-	-	Supported
H_12_	LFE-SES → FRPT → LFET → HE^***^	0.03	0.0071	0.0486	-	-	Supported

95% CI, *p* < 0.001, ^*^FRPT = Mediator, ^**^LFET = Mediator, ^***^FRPT+LFET = Mediator.

The effect of LFE-SES on HE (H_1_) was significant and positive (β = 0.60; *p* < 0.001; LLCI: 0.4914; ULCI: 0.7150). The effect on FRPT (H_2_) was again significant and positive (β = 0.37; *p* < 0.001; LLCI: 0.2846; ULCI: 0.4658), and its effect on the LFET (H_3_) was also significant and positive (β = 0.37; *p* < 0.001; LLCI: 0.2749; ULCI: 0.4670). In addition, while the effect of FRPT on LFET (H_4_) was significant and positive (β = 0.49; *p* < 0.001; LLCI: 0.3977; ULCI: 0.5958), the effect on HE (H_5_) was not significant (β = 0.04; *p* > 0.05; LLCI:−0.0707; ULCI: 0.1702). The effect of LFET on HE (H_6_) was significant and positive (β = 0.16; *p* < 0.05; LLCI: 0.0529; ULCI: 0.2727). When the mediator role of FRPT in the effect of LFE-SES on HE was examined (H_9_), the role of FRPT in this relationship was found to be insignificant (β = 0.01; *p* > 0.05; LLCI:−0.0256; ULCI: 0.0675). Within the scope of the research model, the mediating role of LFET (H_10_) in the effect of LFE-SES on HE was examined, and LFET was shown to be a mediator in this relationship (β = 0.06; LLCI: 0.0178; ULCI: 0.1091). The mediating role (H_12_) of FRPT and LFET in the effect of LFE-SES on HE was examined to test the total model, and statistically significant findings were obtained (β = 0.03; LLCI: 0.0071; ULCI: 0.0486). Accordingly, FRPT and LFET together played a mediating role in the effect of LFE-SES on HE. In line with the results obtained, the mediating role of LFET in the effect of LFE-SES on HE was determined, and the details are shown in Table 9.

**Table 9:** In the effect of LFE-SES on HE; LFET = Mediator role (H_10_), and FRPT → LFET = Mediator role (H_12_).

	**Result variables**			
	**LFET**		**HE**	
	β	**SE**	β	**SE**
LFE-SES (c)			0.71^**^	0.049
*R* ^2^				0.35
LFE-SES (a)	0.37^**^	0.048		
*R* ^2^		0.39		
LFET (b)			0.16^*^	0.055
*R* ^2^				0.37
Total effect (c)				0.71
Indirect Effect (LFET = Mediator)				(H_10_) 0.060; 95% CI (0.0178: 0.1091)
Indirect Effect (FRPT → LFET = Mediator)				(H_12_) 0.030; 95% CI (0.0088: 0.0580)Ô

* *p* < 0.05,

** *p* < 0.001.

These findings indicate that LFET plays a mediating role in the effect of LFE-SES on HE. LFE-SES significantly and positively affects LFET (β = 0.37; *p* < 0.001; LLCI: 0.2749; ULCI: 0.4670). LFE-SES explains LFET by 39% (*R*^2 =^0.39). LFET plays a mediating role at a rate of.06 in the effect of LFE-SES on HE (β = 0.050; *R*^2 =^0.37; LLCI: 0.0148; ULCI: 0.0911). Accordingly, hypothesis H_10_ was supported. The co-mediator role of FRPT and LFET (H_11_) in the effect of LFE-SES on HE was also investigated. The mediator effect was significant and favorable (β = 0.030; *R*^2 =^0.37; LLCI: 0.0088; ULCI: 0.0580). In this instance, the effect of LFE-SES on HE is mediated through FRPT and LFET to a degree of 30%.

## Discussion

### Theoretical implications

The tendency toward healthy eating is strongly and favorably influenced by the impression of social and environmental sustainability in local food experiences. This outcome is in line with several published studies (7, 8, 30, 47–49, 117). According to Kim et al. (7), healthy eating tendencies were positively impacted by individuals' sustainable understanding of themselves. Donato et al. (8) suggested that people develop a sense of healthy eating when food packaging is prepared with sustainability in mind, and Willett et al. (47) argued that research on the creation of sustainable, healthy eating habits would lead to the establishment of new nutritional paradigms. In the context of environmental and social sustainability principles, the 49 discussed healthy eating behaviors and concluded the correlations between these variables. Both models used in our study [Hayes (138) 90th and 6th models] demonstrated the beneficial influence of the perceptions of social and environmental sustainability in the local food experience on healthy eating, and their alignment with current literature was established.

Our study showed that food-related personality traits were positively impacted by the impression of social and environmental sustainability in local food experiences. According to Hopwood et al. (63), personality characteristics and reasons for eating were related. Gustavsen and Hegnes (64) also described a connection between personality traits and preferences for eating organic food that derived from perceptions of sustainability. Identical outcomes have been reported in the literature (153–156). Our findings on the favorable impact of food-related personality traits on perceptions of social and environmental sustainability – found in both model 90 and model 6 – are consistent with previous research.

Research reveals that the perception of social and environmental sustainability in local food experiences positively affects individuals' local food eating tendencies. Individuals' sustainability perception (65) is one of many reasons tourists prefer to eat local food (56). Testa et al. (25) considered individuals' perceptions of social and environmental sustainability among their local food consumption motivations. The relationship between individuals' understanding of sustainability and local food consumption behavior has been the subject of many studies (66, 67, 69–72, 157, 158), and the findings obtained in the present research concur with the literature.

We found a positive effect of food-related personality traits on the tendency to eat local food. Although local cuisine is a tourist attraction (72), only some tourists choose local food. This variability can be explained by individual personality traits (74). Sivrikaya and Pekersen (159) found that food-related personality traits affected tourists' local food eating tendencies. Similarly, Akyuz (160) reported that food-related personality traits played a role in local food consumption motivation, and Pappas et al. (84) stressed the strong relationship between those factors. Again, our research findings showed similarities with the literature.

We found no significant relationship between food-related personality traits and healthy eating tendencies (H_5_). Literature suggests that individuals with a high level of food involvement – a personality trait related to food – prefer a more healthy food (31, 90–92), and Potard (95) described a significant relationship between personality traits and healthy eating. However, the results obtained in the present study did not align with other studies. This may be explained by the challenge of identifying food-based personality traits, particularly neophobia, along with inconsistencies in consumer attitudes that cannot be generalized for political, cultural, and social reasons Faccio and Fovino (161). For instance, consumers with a neophobic attitude do not appreciate alternative dishes, organic food, or regional dishes created to ensure sustainability. However, these individuals may opt to consume foods containing genetically modified organisms (GMOs). This variability may explain the inability to establish a relationship between personality traits based on food and healthy eating. Faria and Kang (162) examined food neophobia and found that physical health motivation was relatively unimportant for individuals and that high levels of food neophobia did not affect eating choices. These findings provide evidence of the power of social factors (e.g., religion and family), and emphasize the role of factors other than health for food choices.

Our study also found that food-related personality traits did not have a mediating role (H_9_) between the perception of social and environmental sustainability in local food experiences and healthy eating. This can be explained by considering that the relationship between personality traits related to food and healthy eating is not significant. At the same time, the results of the research were compared with current literature in an attempt to explain the two unsupported hypotheses (H_5_ and H_9_). Potard (95) incorporated personality traits in his study using the “Big Five” traits theory, focusing on extraversion, agreeableness, conscientiousness, neuroticism, and openness (163). Our study used the “food-related personality traits” theory that associated personality traits with food experience. According to this theory, food-related personality traits include food neophobia, food neophilia, and food involvement (57–59). Additionally, 30 12-item scale and its “food neophobia” and “food involvement” dimensions were used to measure the food-related personality characteristics variable. These differences between studies may account for the distinction between our study and Potard (95) work. In contrast to attitudes toward organic foods, Chen (31) discovered a clinically insignificant link between food neophobia and food preference motive. Individuals with high levels of food involvement were also only found to have positive attitudes toward familiar organic foods, but there was no relationship between their intention to purchase organic or healthy food and their level of food involvement. This partially aligns with the results of this study. Monds et al. (164) focused on the relationship between the personality traits of individuals and their fruit-vegetable consumption behaviors and could not detect a significant relationship between these variables. Likewise, Awad et al. (165) examined the relationship between personality traits and healthy eating at the clinical level, finding a significant relationship between personality traits and healthy orthorexia. In short, the measures assessing personality traits associated with food are insufficient to identify the factors leading to the development of motivations like cultural, political, sociological, health, and food-based personality traits. Our research addressed the relationship between personality traits and healthy eating from several angles, and the findings on the relationship may vary depending on the perspective adopted.

The effect of local food eating tendencies on healthy eating was examined (H_6_). When considered in the 90th model, the relationship was found not significant. However, the 6th model indicated a significant and positive relationship. Model 90 had the online information search variable conditioned as a moderator between local food eating tendencies and healthy eating. However, in model 6, the online information search variable was excluded from the relationship between the two variables – likely explaining the different findings. This situation also emerged in the hypotheses regarding the mediator role of local food eating tendencies. Local food eating tendency – determined as the mediator between the perception of social and environmental sustainability and healthy eating in the local food experience – was insignificant in the 90th model but significant in the 6th model. In the 90th model, the mediating role of the local food eating tendency was examined under the condition of the moderator role of online information searching (H_11_). However, online information searching was not a significant moderator, likely because this situation creates a disadvantage in determining the mediator role of local food eating tendencies. Therefore, the online information search variable was removed from the model, and the hypotheses were retested using model 6, which explained existing relationships more effectively. Thus, the mediator role (H_10_) between the perception of social and environmental sustainability and healthy eating in the food experience of the local food eating tendency was significant in the 6th model. Retesting the relationship in model 6 was important in terms of compatibility with the current literature, given that most studies suggest that local food eating tendency affects healthy eating (38) and that local food is perceived as healthier (36, 37, 39, 40, 69, 97–99).

Local food is related to environmental sustainability because of reduced carbon footprints and social sustainability through promoting local producers. It is also considered healthier than industrial food (133), demonstrating the connection between local eating tendencies, how people view sustainability and healthy eating habits. Yoon et al. (135) suggest that people who frequently choose local food exhibit greater levels of sustainable and healthy eating behaviors. This knowledge highlights the significance of considering sustainability, healthy eating, and local eating tendencies together. From this perspective, the study findings overlap with the existing literature.

Our study examined the moderating role of the online information search variable in some relationships based on the 90th model. The moderating role of online information search in the assumed relationship between the perception of social and environmental sustainability and healthy eating in the food experience (H_7_) and between the local food eating tendency and healthy eating (H_8_) was found to be insignificant. In addition, online information searching (H_11_) was not a significant moderator in the conditional indirect effect of the perception of social and environmental sustainability in local food experiences on healthy eating, in which local food eating tendency plays a mediator role. Literature suggests that online information search – chosen as a moderator variable – was not related to the independent factors but rather to healthy eating. Although this presumption is consistent with the literature (101–103), the research was unable to support it. The online environment is an intensive source of knowledge and a valuable tool for learning about healthy eating (107–109). Social media also aids in raising public awareness of healthy eating (115). Online information search was important for people who wanted to transform their eating habits and become healthier. Based on this knowledge, we assumed that online information search would have a moderating role in the interactions. However, the moderating impact was negligible, compelling the researchers to determine the underlying reasons. Reviews of studies focusing on healthy eating and information search practices have been conducted. Lee et al. (166) found no connection between healthy eating practices and traditional or digital information search methods. Overall, there is insufficient empirical evidence on the moderating role of online information search in these relationships, a problem further compounded by the different theoretical perspectives used in studies. Furthermore, our study found no statistical evidence indicating that searching for information online was directly related to healthy eating.

The results showed that food-related personality traits and local food eating tendencies have a mediator role (H_12_) at a rate of 0.03 (model 6) in the relationship between the perception of social and environmental sustainability in local food experiences and healthy eating. Although the mediator role of food-related personality traits did not reach significance in either model, when evaluated together with the tendency to eat local food, a significant mediator role emerged in the total model (model 6). The rate of the tendency toward healthy eating was *R*^2^ = 0.37. This important finding provides a fundamental explanation for healthy eating and is a strength of the research within the framework of the tested models. From this perspective, the results of our study elucidate preferences for healthy eating. We reported a positive and substantial association between the preference for local eating and the perception of social and environmental sustainability in the local food experience and food-related personality traits. Our study makes a significant theoretical contribution to the literature by providing the disclosure rate of local food eating tendencies (*R*^2^ = 0.39).

### Practical implications

The findings from our study have practical implications concerning the local food phenomenon. Our findings are important for comprehending healthy eating trends in the context of local foods. The main factor influencing this outcome is how consumers perceive social and environmental sustainability in their local food experiences. Attitudes toward environmental sustainability and healthy eating should be developed early in life when, according to studies of preschoolers, sustainable and healthy eating behaviors are adjustable (167–169). Thus, insights from the study could inform interventions applied from a young age. Fabri et al. (170) reviewed the food-based dietary guidelines of several countries, observing that sustainability and healthy eating are insufficiently incorporated in many of them – an important issue that policymakers should consider. In terms of reducing environmental impact, producing plant-based foods (48, 49) is essential for future nutrition models. Local food producers should be aware of this and develop their production policies accordingly. Local cuisine practices are viewed as a tool for social development by the 49, demonstrating the need for decision-makers and policymakers to support local cuisine in all forms. Sustainable practices in the context of locally grown food are predicted to raise awareness of healthy diets. This knowledge is crucial for local producers' marketing strategies. According to Donato et al. (8), consumers are more likely to perceive items as being healthy because of the sustainable techniques implemented in their packaging. Our findings support a significant recommendation for the industry: local product packaging methods should incorporate messages regarding recycling, environmental friendliness, and local market support.

Sustainable practices in local food experiences positively affect individuals' tendencies to eat local food. Such practices applied within the framework of local food make individuals eager for local food or increase their existing desires. This finding has important implications for local food producers and marketers. Alsetoohy et al. (67) regarded the purchase of local food as a sustainable behavior. This knowledge, evaluated especially in terms of social sustainability, should be considered by companies serving tourist groups. Tour companies should encourage tourist groups to buy local products, clearly emphasizing that this occurs in the context of sustainability.

Local food eating tendencies increase individuals' healthy eating intentions. Individuals with a high local food eating tendency expect local products to be natural and healthy. These foods should be produced in more transparent, fresh, and additive-free forms (69). Local product outlets should encourage consumers to adopt better nutrition or healthier diets, and these practices should be explicitly incorporated into marketing activities. In summary, sustainable, local, and healthy practices should be considered by the relevant sectors and decision-makers because they play a significant role in attracting the attention of tourists.

### Limitations and future research

The study has some limitations. The first is using a cross-sectional design, which constrained the interpretation of the findings. Future research using longitudinal approaches would enable greater generalization of the findings and overcome this restriction. Second, we used a Turkish sample to gather research data, but future research could be designed to reflect economic, social, and cultural diversity in other countries, comparing the results with the results we obtained here. Third, the mediator role of food-related personality traits between the perception of social and environmental sustainability in local food experiences and healthy eating was found to be insignificant. In our study, personality traits were measured using the concept of food-related personality traits, and some insignificant results were obtained. In the future, measurements made in the context of other personality trait theories may elucidate these findings and the relationships between variables. Fourth, the research model showed that online research had no moderating effect on healthy eating habits. Our study used a categorical yes/no question to gauge participants' level of online information search. Future research could use a more specific measurement approach to obtain more detailed results. Fifth is the disclosure rate of the healthy eating variable. Other psychological variables could be revealed besides those used in the model we tested here. Thus, the rate of explanation of variables might be increased with new models.

## Conclusions

This study focused on the effect of food experience perceptions of social and environmental sustainability on healthy eating. The purpose was to identify local food eating tendencies and food-related personality factors that mediate this effect and the moderating effects of online information search. A research model was tested using Hayes (138) PROCESS Macro model 90. The analyses revealed that online information search – selected as a moderator variable – was inconsequential in every instance, and other relationships in the model were affected by this. Thus, Hayes (138) PROCESS Macro model 6 was used to restructure the research model (2 mediators). The findings suggested that local food eating tendencies and personality factors associated with food function as mediators in tandem. When the variables were assessed separately, the mediator effect of local food eating tendency was substantial, and the impact of food-related personality traits was minor. Our study makes theoretical and practical contributions to advance knowledge of local food experiences and sustainable, healthy eating.

## Data availability statement

The original contributions presented in the study are included in the article/Supplementary material, further inquiries can be directed to the corresponding author.

## Ethics statement

The studies involving human participants were reviewed and approved by the Atatürk University Social and Human Sciences Ethics Committee (Ref: 2023:1-14). The patients/participants provided their written informed consent to participate in this study.

## Author contributions

Conceptualization, software, writing—original draft, writing—review and editing, visualization, and project management: ZA, SO, SHB, and YB. Methodology and data curation: SO, SHB, and YB. Formal analysis and surveillance: SO and YB. References: SO. All authors have read and accepted the published version of the article.
