# Supplementary material for: Effect of perception of sustainability in local food experiences on healthy eating tendency: mediator and moderator effects
Source: Front Nutr. 2023 Jun 7;10:1150277. doi: 10.3389/fnut.2023.1150277 (PMC10283074; doi:10.3389/fnut.2023.1150277)
Supplement: Supplementary file 1 [file Data_Sheet_1.docx]

Supplementary Material

Effect of Perception of Sustainability in Local Food Experiences on Healthy Eating Tendency: Mediator and Moderator Effects

Zühal AKSAKALLI BAYRAKTAR*, Serhan ORAL, Samuray Hakan BULUT, Yusuf BAYRAKTAR

*** Correspondence:** Zühal AKSAKALLI BAYRAKTAR: [zuhal.aksakalli@atauni.edu.tr](mailto:zuhal.aksakalli@atauni.edu.tr)

# Supplementary Figures and Tables


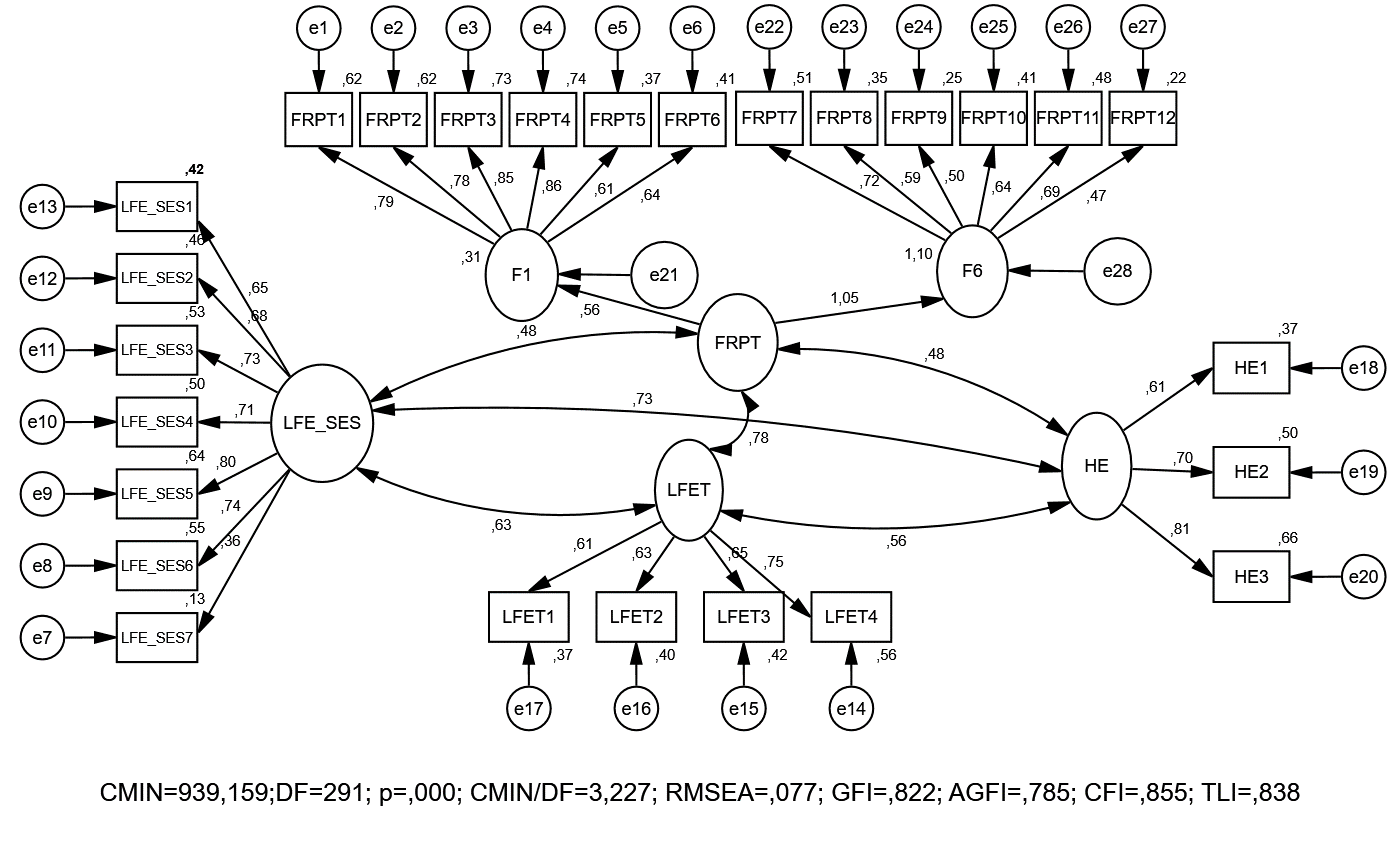


**Supplementary Figure 1.** Measurement Model 1


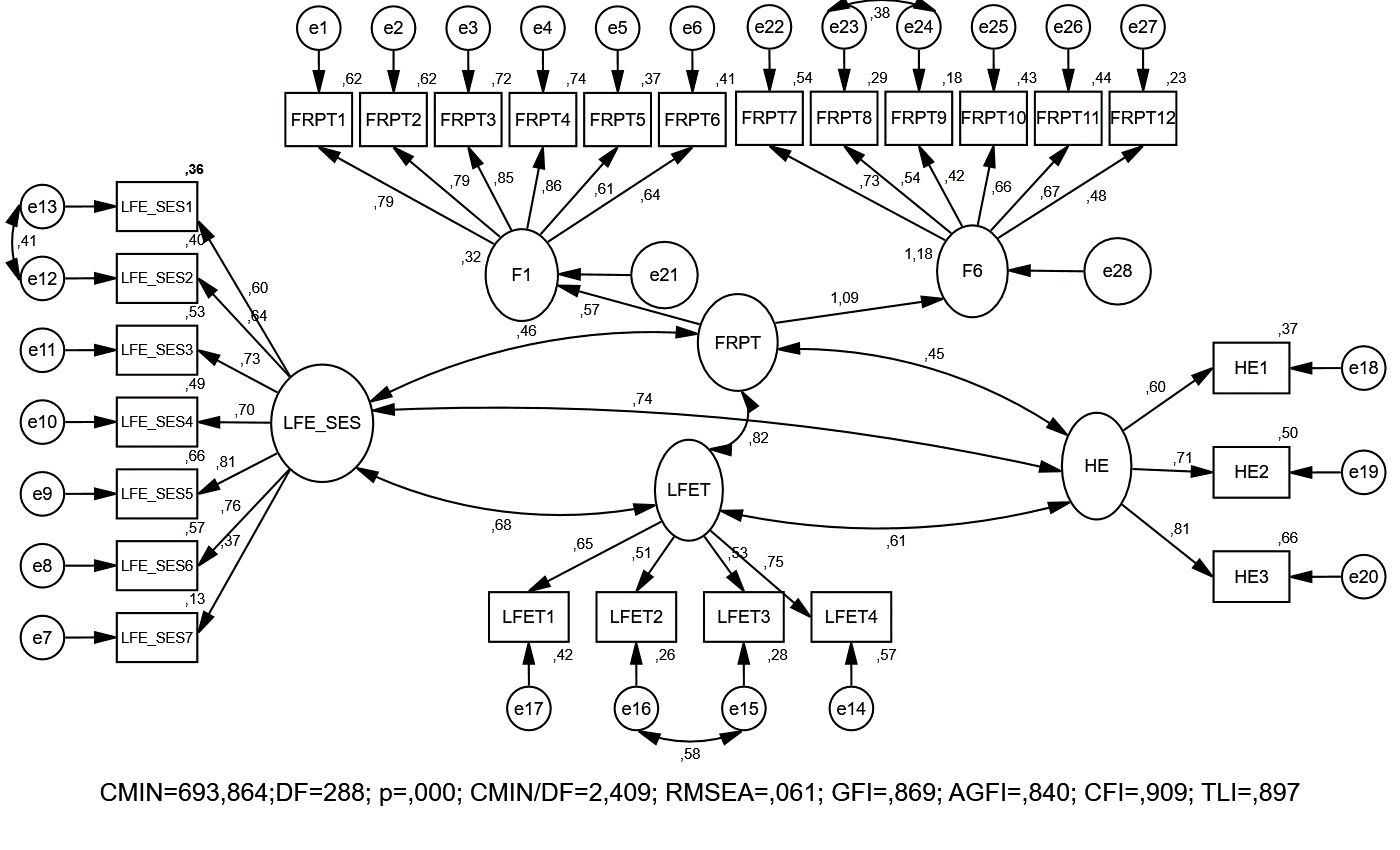


**Supplementary Figure 2.** Measurement Model 2

**Supplementary Table 1.** Measurement Model Results

|  |  | β^0^ | β^1^ | SE | Test. Sta. | p | R^2^ |
| --- | --- | --- | --- | --- | --- | --- | --- |
| Eating local food allows me to be in solidarity with local farmers | LFE_SES1 | ,603 | 1,263 | ,199 | 6,332 | **<0,001** | ,617 |
| Eating local food allows me to contribute to the local economy | LFE_SES2 | ,636 | 1,307 | ,203 | 6,443 | **<0,001** | ,265 |
| Eating local food allows me to contribute to maintaining agricultural landscape | LFE_SES3 | ,729 | 1,645 | ,246 | 6,694 | **<0,001** | ,418 |
| I like to eat local food because it has not travelled long distances and is therefore more  environmentally sustainable. | LFE_SES4 | ,702 | 1,450 | ,219 | 6,630 | **<0,001** | ,366 |
| Eating local food I contribute to conserving the environment and its natural resources | LFE_SES5 | ,813 | 1,791 | ,261 | 6,859 | **<0,001** | ,499 |
| Local food are more environmentally-friendly | LFE_SES6 | ,755 | 1,868 | ,277 | 6,751 | **<0,001** | ,662 |
| It is important to me that local food I eat is organic certified | LFE_SES7 | ,365 | 1,000 |  |  |  | ,620 |
| I am constantly sampling new and different foods | FRPT1 | ,787 | 1,000 |  |  |  | ,230 |
| I do not trust new foods | FRPT2 | ,785 | 1,001 | ,061 | 16,394 | **<0,001** | ,444 |
| If I do not know what is in a food, I won’t try it | FRPT3 | ,851 | 1,385 | ,076 | 18,107 | **<0,001** | ,430 |
| I am afraid to eat things I have never had before | FRPT4 | ,860 | 1,282 | ,070 | 18,340 | **<0,001** | ,177 |
| I will eat almost anything | FRPT5 | ,609 | 1,012 | ,084 | 12,110 | **<0,001** | ,287 |
| Cooking or barbequing is not much fun | FRPT6 | ,639 | ,768 | ,060 | 12,787 | **<0,001** | ,536 |
| Talking about what I ate or am going to eat is something I like to do | FRPT7 | ,732 | 1,000 |  |  |  | ,571 |
| When I travel, one of the things I anticipate most  is eating the food there | FRPT8 | ,536 | ,802 | ,084 | 9,573 | **<0,001** | ,133 |
| I do most or all of the clean up after eating | FRPT9 | ,421 | ,710 | ,095 | 7,508 | **<0,001** | ,408 |
| I enjoy cooking for others and myself | FRPT10 | ,656 | ,617 | ,053 | 11,675 | **<0,001** | ,371 |
| I do most or all of my own food shopping | FRPT11 | ,666 | 1,021 | ,086 | 11,855 | **<0,001** | ,740 |
| I care whether or not a table is nicely set | FRPT12 | ,479 | ,727 | ,085 | 8,577 | **<0,001** | ,724 |
| I prefer to eat at places where local people eat | LFET1 | ,647 | ,912 | ,078 | 11,674 | **<0,001** | ,405 |
| I would like to participate in local cuisine courses | LFET2 | ,514 | 1,004 | ,109 | 9,244 | **<0,001** | ,364 |
| I want to prepare food and beverages that I have tried in the places I have visited | LFET3 | ,533 | ,947 | ,099 | 9,591 | **<0,001** | ,569 |
| I would like to experience food and beverages in places where offered according to local traditions | LFET4 | ,754 | 1,000 |  |  |  | ,284 |
| Knowing the producer is for me a guarantee of the wholesomeness of local food | HE1 | ,605 | 1,000 |  |  |  | ,532 |
| I believe that the local food is free of synthetic chemicals that are harmful to health | HE2 | ,707 | 1,260 | ,123 | 10,208 | **<0,001** | ,493 |
| I prefer to consume local food because it is good for health | HE3 | ,813 | 1,301 | ,120 | 10,836 | **<0,001** | ,661 |
